# Upstream stimulatory factor 2 (USF2) induced upregulation of triggering receptor expressed on myeloid cells 1 (TREM1) promotes endometritis by regulating toll-like receptor (TLR) 2/4-nuclear factor-kappaB (NF-κB) signaling pathway

**DOI:** 10.1080/21655979.2022.2030619

**Published:** 2022-01-31

**Authors:** Miao Zhang, Chengkun Yin, Yan Chen, Juan Wang, Jing Jiang

**Affiliations:** a Department of Gynecology and Obstetrics, The Affiliated Hospital of North Sichuan Medical College, Nanchong, China; b Department of Radiology, Suining Central Hospital, Suining, China

**Keywords:** Endometritis, TREM1, USF2, TLR2/4, NF-κB

## Abstract

Triggering receptor expressed on myeloid cells 1 (TREM1) participates in the development of endometritis. This study aims at identifying the effects and interaction of TREM1 and upstream stimulatory factor 2 (USF2) in endometritis by using a model of lipopolysaccharide (LPS)-induced human endometrial epithelial cells (HEnEpCs). ELISA was performed to determine the levels of interleukin (IL)-6, IL-1β, and tumor necrosis factor (TNF-α) after LPS stimulation. TREM1 and USF2 expression was examined with RT-qPCR and Western blot. The JASPAR database was employed to predict the binding site between USF2 and TREM1, which was confirmed by luciferase reporter and chromatin immunoprecipitation assays. After TREM1 overexpression, IL-6, IL-1β, and TNF-α expression was detected by ELISA. Next, the binding of TREM1 to toll-like receptor (TLR) 2/4 was examined with co-immunoprecipitation. Then, proteins in TLR2/4-nuclear factor-kappaB (NF-κB) signaling in HEnEpCs under LPS condition were assessed by Western blot or immunofluorescence before and after TREM1 knockdown. Finally, TLR2 or TLR4 was silenced to explore whether intervene TLR2/4-NF-κB signaling pathway could rescue TREM1-overexpression-induced inflammation in LPS-induced HEnEpCs. Results revealed that upregulated TREM1 was observed in LPS-challenged HEnEpCs. Next, USF2 was found to have transcriptionally active TREM1 expression. Additionally, USF2 knockdown decreased the levels of IL-6, IL-1β, and TNF-α, whereas this effect was rescued after TREM1 overexpression. Besides, TREM1 could bind to TLR2/4 to regulate NF-κB signaling. Moreover, the intervention of TLR2/4-NF-κB signaling pathway rescued TREM1-overexpression-induced inflammation in LPS-stimulated HEnEpCs. Collectively, USF2 promotes endometritis by upregulating TREM1, thereby activating TLR2/4-NF-κB pathway.

## Introduction

Endometritis is an inflammation-induced change in the structure of the endometrium [1]. This common gynecological disease is mainly manifested as fever, lower abdominal pain, leucorrhea abnormality, foul odor, and so on [2]. Bacteria can travel up the vagina, cervix, or down the fallopian tubes and through the lymphatic system to reach the endometrium [3]. If the uterine cavity is in a good drainage condition and can achieve monthly shedding of the endometrial lining, there is only a slim chance of long-term bacterial infection in the endometrium. However, reoccurrence of inflammation is expected if the treatment is incomplete in the acute stage of endometritis or if the source of infection frequently exists. In severe cases, endometritis can affect the myometrium and eventually grow into myositis [4]. Endometritis can be divided into acute endometritis and chronic endometritis [5], among which chronic endometritis often coexists with chronic cervicitis and chronic salpingitis and is the most common cause of miscarriage [6,7]. Effective targeting and prevention of recurrent inflammation in endometritis is considered a direct treatment for this disease.

Triggering receptor expressed on myeloid cells 1 (TREM1) was identified fairly recently around 20 years ago and is described as an activating receptor of the immune globulin superfamily expressed on human myeloid cells, including neutrophils, macrophages, and endothelial cells [8]. Studies have corroborated its implication in the immune response involving proinflammatory chemokine interleukin (IL)-8 and proinflammatory cytokine tumor necrosis factor (TNF-α) [9,10]. More importantly, a recent study has reported that knocking out TREM1 may have the ability to inhibit lipopolysaccharide (LPS)-induced inflammatory response in an endometritis model with a highly pathogenic LPS infection in mice uteri [11]. However, the action mechanism underlying this potential effect remains to be elucidated.

According to the prediction on PROMO database (http://alggen.lsi.upc.es/cgi-bin/promo_v3/promo/promoinit.cgi?dirDB=TF_8.3), upstream stimulatory factor 2 (USF2) could be a transcription factor of TREM1. In addition, USF2 has been found to be upregulated in endometriotic stromal cells [12]. Therefore, we presumed that USF2 might transcriptionally activate TREM1 to promote endometritis. Additionally, by using the Search Tool for the Retrieval of Interacting Genes/Proteins (STRING; http://string-db.org/cgi/input.pl) database, we predicted that TREM1 may interact with Toll-like receptors (TLR) 2 and TRL4. Compelling evidence also indicate that TREM1-deletion decreases inflammation in spinal cord tissue of mice with spinal cord injury by inhibiting TLR2/4-NF-κB signaling [13]. The suppression of TLR2/4-mediated NF-κB activation can relieve endometritis [14,15].

In the present study, an in vitro model of LPS-induced human endometrial epithelial cells (HEnEpCs) was established to investigate whether USF2 can induce transcriptional activation of TREM1 and promote endometritis by regulating TLR2/4-NF-κB signaling pathway. Our findings might identify a novel target for the treatment of endometritis.

## Materials and methods

### Cell culture and treatment

Human endometrial epithelial cells (HEnEpCs) were acquired from Lifeline Cell Biotechnology. Cells were grown in a human endometrial cell growth medium (Cell Applications; San Diego, CA, USA) supplemented with 10% fetal bovine serum (FBS; AlphaCell, Shenzhen, China) in a humidified incubator with 5% CO_2_ at 37°C. LPS (Sigma-Aldrich, St. Louis, MO, USA) at a concentration of 1 μg/mL was chosen to stimulate the cells for 24 h [16].

### Cell transfection

Small hairpin RNA (shRNA) targeting USF2 (shUSF2), TREM1 (shTREM1), TLR2 (shTLR2), or TLR4 (shTLR4); the scrambled negative control (sh-NC); pcDNA3.1 empty vector (pcDNA 3.1); and pcDNA3.1 plasmid of TREM1 (pc-TREM1) were constructed by GenePharma (Shanghai, China). They were, respectively, transfected into HEnEpCs for different groups using Lipofectamine 3000 (Invitrogen; Thermo Fisher Scientific, Inc.). Successful transfection was evaluated with reverse transcription-quantitative polymerase chain reaction (RT-qPCR) or Western blot analysis at 48 h after transfection.

### Enzyme-linked immunosorbent assay (ELISA)

ELISA was performed to detect the levels of proinflammatory cytokines in LPS-challenged HEnEpCs. After collection of the cell supernatant, the levels of interleukin IL-6, IL-1β, and TNF-α were measured employing ELISA kits (Shanghai XiTang Biotechnology Co., Ltd., Shanghai, China) in accordance with the manufacturer’s protocol. The optical density values were read using a plate reader (BioTek Instruments, Inc.).

### Dual-luciferase reporter assay

Luciferase reporter plasmids (Promega Corporation) were constructed with the wild-type TREM1 promoter (WT-TREM1) or mutant TREM1 promoter (Mut-TREM1). The luciferase reporter plasmids and USF2-expressing plasmid were co-transfected into the HEnEpCs cells using Lipofectamine 3000 reagent (Invitrogen; Thermo Fisher Scientific, Inc.). The luciferase activity was detected by means of the Dual-Luciferase Assay System (Promega) at 48 h after transfection.

### Chromatin immunoprecipitation (ChIP)

Proteins and DNA of the cells were first cross-linked by 0.75% paraformaldehyde (Aladdin, Shanghai, China). The cells were then lysed with sodium dodecyl sulfate (SDS) lysis buffer (Beyotime, Nanjing, China) on ice for 10 min. The lysate was ultrasonically treated to break the DNA into fragments of about 500–1000 bp. USF2 antibody and IgG antibody were added to the chromatin for immunoprecipitation. DNA was eluted and purified at last. PCR was performed to detect the relative enrichment of TREM1.

### Co-immunoprecipitation (Co-IP)

Cells in the culture plate were first washed with phosphate buffer and were scraped off into 1 mL of cold lysis buffer for IP (Beyotime). After the cell suspension was centrifuged, anti-TREM1 (ab214202; Abcam, Cambridge, MA, USA), anti-TLR2 (ab209216; Abcam, Cambridge, MA, USA), anti-TLR4 (ab22048; Abcam, Cambridge, MA, USA), and control lgG for 1 µg plus Protein A/G beads (Santa Cruz Biotechnology, CA, USA) were, respectively, added to the supernatant for co-immunoprecipitation. The beads were washed with phosphate-buffered saline (PBS) three times, and the immunoprecipitants were assessed via Western blot.

### Immunofluorescence (IF)

IF was performed to determine phospho (p)-NF-κB p65 expression. After transfection of shTREM1, LPS-challenged HEnEpCs on the coverslip were washed three times with PBS and fixed with 4% paraformaldehyde for 10 min. Subsequently, the coverslip was incubated with primary antibody and fluorescein isothiocyanate (FITC)-labeled secondary antibody, respectively, for 1 h at room temperature. 4’,6-Diamidino-2-phenylindole (DAPI) was used to stain the cells for 10 min before the observation of p-NF-κB p65 expression level with a fluorescence microscope (Olympus Corporation).

### RT-qPCR assay

Total RNA was isolated from the cells with the use of TRIzol® (Thermo Fisher Scientific, Inc.) and was reversely transcribed into first-strand complementary DNA (cDNA) employing the Universal RT-PCR Kit (Solarbio, Beijing, China). qPCR was performed on the ABI 7500 PCR system (Applied Biosystems; Thermo Fisher Scientific, Inc.). Glyceraldehyde-phosphate dehydrogenase (GAPDH) was used as the control. Relative mRNA expression was normalized and calculated with the 2^−ΔΔCq^ method [17].

### Western blot analysis

Total proteins were extracted from the cells using a lysis buffer (Beyotime). After denaturation of the proteins, electrophoresis was performed using a 10% sodium dodecyl sulfate-polyacrylamide gel electrophoresis (SDS-PAGE) gel. Subsequently, the proteins were transferred to a polyvinylidene fluoride (PVDF) membrane (Merck Millipore, Billerica, MA, USA) and immersed into 5% skimmed milk for 2 h at room temperature. The membrane was then incubated with specific primary antibodies overnight at 4°C followed by horseradish peroxidase (HRP)-conjugated secondary antibody (Cell Signaling Technology, Danvers, MA, USA) for 1.5 h at room temperature. Images of the protein bands were developed using an enhanced chemiluminescence (ECL) detection system (Applygen Technologies, Inc.). GAPDH was used as the internal control.

### Statistical analysis

All assays in this study were performed in triplicate. Data were analyzed with GraphPad Prism 8.0 and are expressed as the mean ± standard deviation (SD). Student’s t-test was chosen to compare the differences between the two groups. One-way analysis of variance (ANOVA) followed by Tukey’s post hoc test was performed to compare the statistical differences among different groups. P < 0.05 represents significant difference.

## Results

### TREM1 is upregulated in LPS-induced HEnEpCs

**Figure 1.**
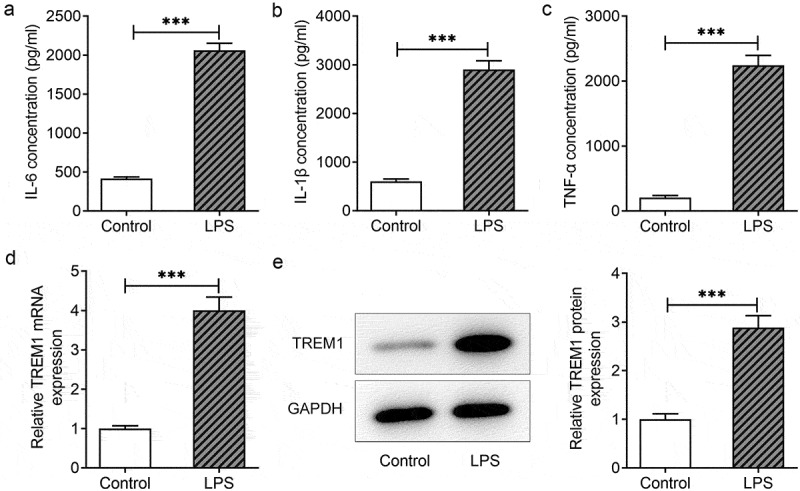
*TREM1 was unregulated in LPS-induced HEnEpCs*. (a–c) The concentrations of proinflammatory cytokines IL-6, IL-1β, and TNF-α in LPS-induced HEnEpCs, detected by ELISA. (d–e) The mRNA and protein expression of TREM1 in LPS-challenged HEnEpCs, detected by RT-qPCR and Western blot assay. ***P < 0.001.

TREM1 deficiency has been reported to inhibit LPS-induced inflammatory response in an endometritis model with a highly pathogenic LPS infection in mice uteri [11]. This study is the first to explore the role of TREM1 in HEnEpCs exposed to LPS. After LPS stimulation in HEnEpCs, the expression of proinflammatory IL-6, IL-1β, and TNF-α was found to be considerably elevated in contrast with the control group (Figure 1(a-c)). Moreover, both RT-qPCR and Western blotting detected increased expression of TREM1 in HEnEpCs following LPS stimulation (Figure 1(d-e)). It is thus suggested that LPS induces inflammatory response and TREM1 upregulation in HEnEpCs.

### USF2 transcriptionally activates TREM1 in LPS-induced HEnEpCs

**Figure 2.**
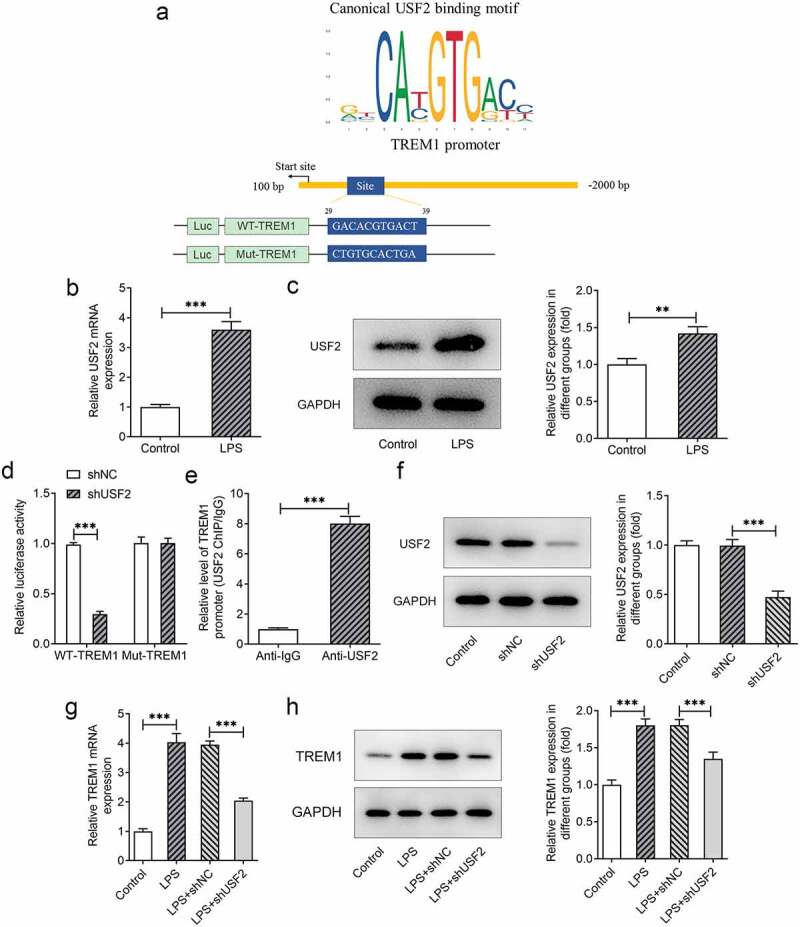
*TREM1 was transcriptionally activated by USF2 in LPS-induced HEnEpCs*. (a) The TREM1 promoter region that could to be bound by USF2 was predicated by JASPAR database. (b–c) The mRNA and protein expression of USF2 in LPS-challenged HEnEpCs, detected by RT-qPCR and Western blotting. (d) Relative luciferase activity in the group of WT-TREM1 promotor + shNC, WT-TREM1 promotor + shUSF2, Mut-TREM1 promotor + shNC, or Mut-TREM1 promotor + shUSF2, detected by dual-luciferase reporter assay. (e) Relative enrichment of TREM1 promoter in the presence of anti-USF2, detected by ChIP assay. (f) USF2 expression before and after transfection of shUSF2, detected by Western blot. (g–h) The mRNA and protein expression of TREM1 before and after USF2 knockdown in LPS-challenged HEnEpCs, detected by RT-qPCR and Western blot. **P < 0.01; ***P < 0.001.

To investigate the potential regulatory mechanism of TREM1 in LPS-induced HEnEpCs, JASPAR database was used to predict the possible transcription factors that could regulate TREM1. USF2 was found to be a transcription factor for TREM1 by binding to TREM1 promoter region (29 ~ 39) (Figure 2(a)). Additionally, USF2 was also found to be overexpressed in LPS-challenged HEnEpCs (Figure 2(b-c)). Results of the luciferase reporter assay showed reduced luciferase activity in the WT-TREM1 promotor + shUSF2 group as compared to the WT-TREM1 promotor + shNC group (Figure 2(d)). ChIP assay further demonstrated a much higher level of TREM1 promotor in the anti-USF2 group (Figure 2(e)). Then, we transfected shUSF2 into the cells and knocked down its expression (Figure 2(f)). A notable decrease in TREM1 expression was first observed after USF2 knockdown in LPS-induced HEnEpCs in comparison to the NC group (Figure 2(g-h)). These results confirm that USF2 is a transcription factor for TREM1 and that it transcriptionally activates TREM1 in LPS-challenged HEnEpCs.

### TREM1 overexpression reverses the effect of USF2 knockdown on LPS-induced inflammation of HEnEpCs

**Figure 3.**
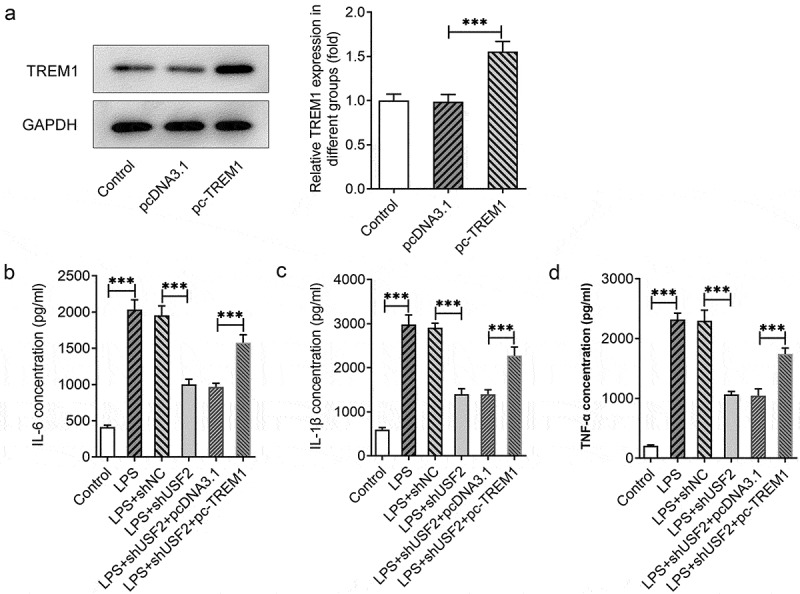
*TREM1 overexpression reversed the anti-inflammatory effect of USF2 knockdown on HEnEpCs stimulated by LPS*. (a) TREM1 expression before and after transfection of pc-TREM1, detected by Western blot. (b–d) The concentrations of proinflammatory cytokines IL-6, IL-1β, and TNF-α before and after USF2 knockdown in the presence or absence of TREM1 overexpression in LPS-challenged HEnEpCs, detected by ELISA. ***P < 0.001.

We then transfected pc-TREM1 and/or shUSF2 into the cells to further identify the effects of USF2 knockdown on LPS-triggered inflammation in HEnEpCs and whether TREM1 upregulation could affect this impact. The result of Western blot illustrated TREM1 overexpression in LPS-challenged HEnEpCs after transfection of pc-TREM1 (Figure 3(a)). According to the results of ELISA (Figure 3(b-d)), the levels of proinflammatory cytokines IL-6, IL-1β, and TNF-α in LPS-challenged HEnEpCs were decreased by USF2 knockdown, which were, however, rescued after TREM1 overexpression. These results suggest that the inhibitory effect of USF2 knockdown on LPS-induced inflammatory response in HEnEpCs can be rescued by overexpressing TREM1.

### TREM1 directly interacts with TLR2/4 in LPS-challenged HEnEpCs

**Figure 4.**
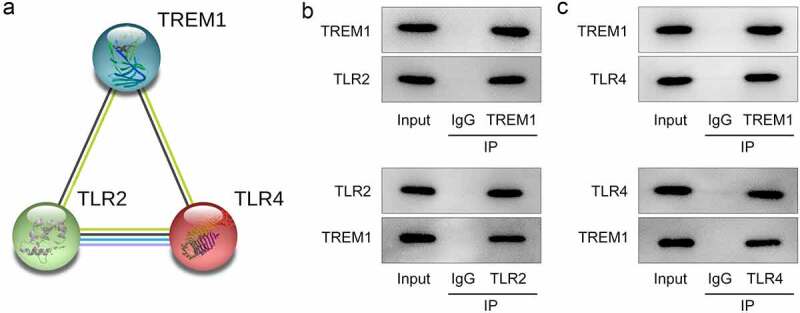
*TREM1 could direct interact with TLR2/4*. (a) Prediction on STRING database of TREM1 interacting with TLR2/4. (b–c) Verification of the interacting between TREM1 and TLR2/4 by co-IP assay.

To further explore the possible mechanism of TREM1 in the regulation of HEnEpCs stimulated by LPS, the STRING database was employed to analyze the genes that could be interacted with TREM1. It was found that TREM1 may interact with TLR2 and TRL4 (Figure 4(a)). Results of Co-IP assay indicated that TREM1 co-immunoprecipitated with both TLR2 and TLR4 (Figure 4(b-c)). These findings validate STRING prediction that TREM1 can directly interact with TLR2/4.

### TREM1 regulates TLR2/4-NF-κB signaling pathway in LPS-induced HEnEpCs

**Figure 5.**
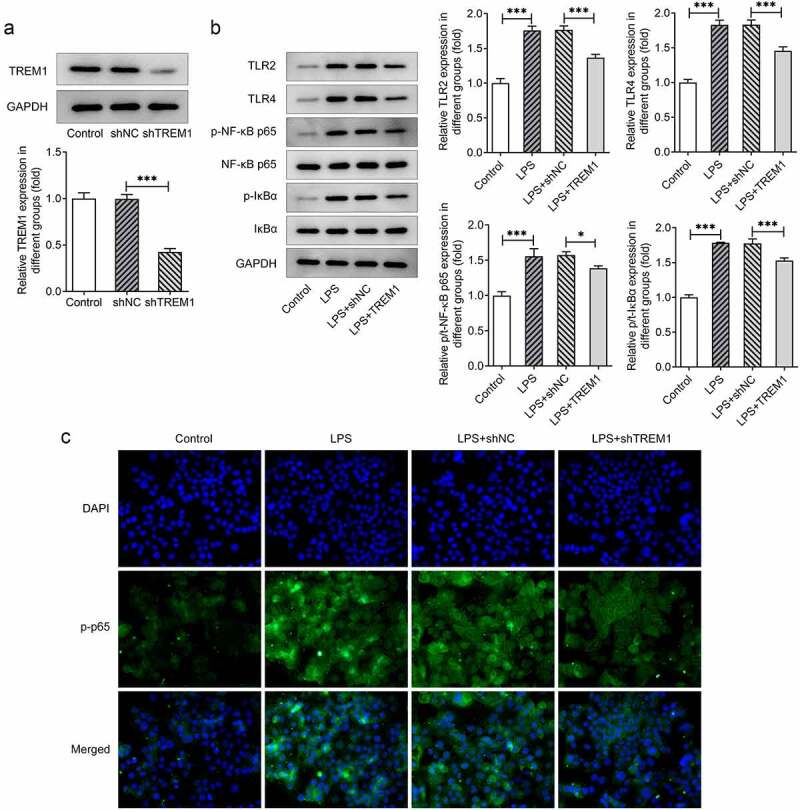
*TREM1 inhibited TLR2/4-NF-κB signaling pathway in LPS-induced HEnEpCs*. (a) TREM1 expression before and after transfection of shTREM1, detected by Western blot. (b) The expression of TLR2, TLR4, p-NF-κB p65, and p-IκBα before and after TREM1 knockdown in LPS-challenged HEnEpCs, detected by Western blot. (c) The fluorescent level of p-NF-κB p65 before and after TREM1 knockdown in LPS-challenged HEnEpCs, detected by IF assay. *P < 0.05; ***P < 0.001.

The subsequent experiments examined whether TREM1 could affect TLR2/4-NF-κB signaling pathway in HEnEpCs exposed to LPS. As presented in Figure 5(a), TREM1 expression was remarkably downregulated after transfection with shTREM1 relative to the shNC group. Then, the expression of TLR2 and TLR4 and that of their downstream NF-κB in LPS-challenged HEnEpCs after TREM1 deletion was tested by using Western blot analysis. As LPS induced increased expression of TLR2, TLR4, p-NF-κB p65, and p-inhibitor of nuclear factor-κB-α (IκB-α), TREM1 knockdown significantly decreased their expression in LPS-challenged HEnEpCs (Figure 5(b)). Meanwhile, p-NF-κB p65 also exhibited stronger fluorescence after LPS stimulation, which was largely inhibited by TREM1 knockdown (Figure 5(c)). The above results indicate that TREM1 can inhibit TLR2/4-NF-κB signaling pathway in LPS-challenged HEnEpCs.

### Intervention of TLR2/4-NF-κB signaling pathway rescues TREM1-overexpression-induced inflammation in LPS-stimulated HEnEpCs

**Figure 6.**
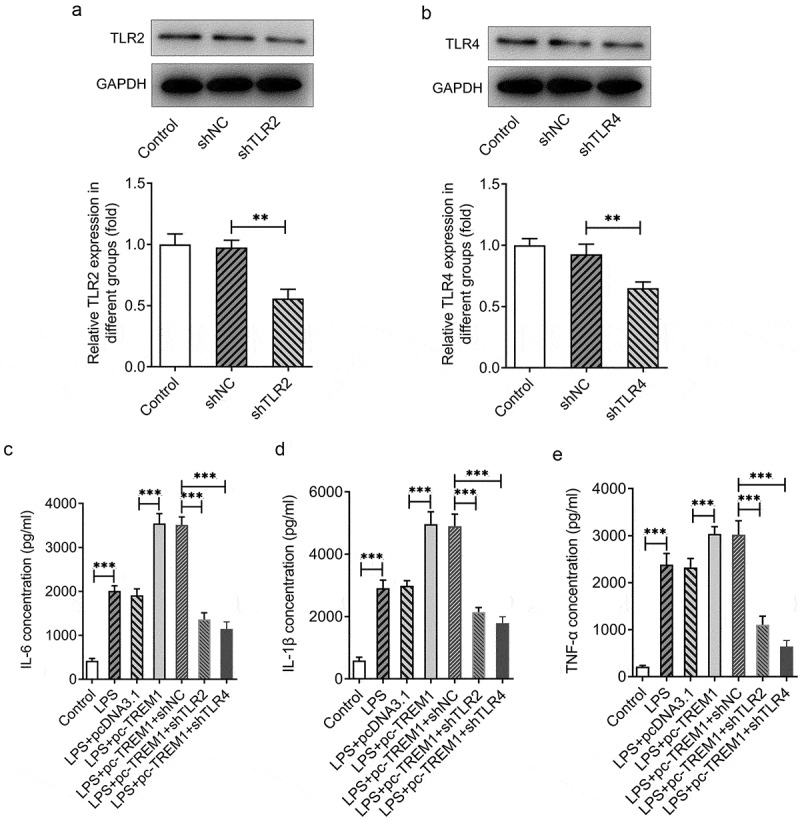
*Intervention of TLR2/4-NF-κB signaling pathway rescues TREM1-overexpression-induced inflammation in LPS-stimulated HEnEpCs*. The expression of (a) TLR2 and (b) TLR4 after transfection with shTLR2 and shRLR4 was tested by Western blot analysis. (b–d) The concentrations of proinflammatory cytokines IL-6, IL-1β, and TNF-α were detected by ELISA kits. **P < 0.01; ***P < 0.001.

To further explore whether intervene TLR2/4-NF-κB signaling pathway could rescue TREM1-overexpression-induced IL-6, IL-1β, and TNF-α in LPS-challenged HEnEpCs, TLR2 or TLR4 was silenced by transfection with shTLR2 or shTLR4. As presented in Figure 6(a-b), TLR2 and TLR4 expression was significantly downregulated in the shTLR2 and shTLR4 groups when compared to the shNC group. It could then be found that TREM1 overexpression notably elevated IL-6, IL-1β, and TNF-α concentrations as compared to the LPS+pcDNA3.1 group (Figure 6(c-d)). Importantly, shTLR2 or shTLR4 silencing, respectively, attenuated TREM1-overexpression-induced IL-6, IL-1β, and TNF-α increase in LPS-stimulated HEnEpCs. These results suggest that the intervention of TLR2/4-NF-κB signaling pathway rescues TREM1-overexpression-induced inflammation in LPS-stimulated HEnEpCs.

## Discussion

Endometritis is a multifactorial inflammatory disease that occurs on the site of the endometrium, potentially jeopardizing the health of both humans and animals [18]. Endometritis can be classified into two categories: acute and chronic [19]. Among the many factors, bacterial invasion is considered a common cause for the development and reoccurrence of endometritis. Gram-negative *Escherichia coli* (*E. coli*) is the main pathogenic microorganism involved in the progression of this disease [20]. LPS as a product of *E. coli* acts as the predominant virulence factor in endometritis, resulting in a severe inflammatory response [21]. In view of its easy relapse and extensive influence on the global population, developing a more effective strategy is in urgent need for the treatment of endometritis [22,23].

TREM and TREM-like receptors are linked to the inflammatory response in both innate immunity and adaptive immunity, as reviewed previously [24]. TREM1 was initially found to play a crucial part in septic inflammation and was later identified by mounting evidence as a mediator of inflammatory response in various diseases including inflammatory bowel disease and spinal cord injury [13,25,26]. Recently, a study has reported a positive association between TREM1 and LPS-induced inflammatory response in mouse model of endometritis [11]. However, the study included no description of the underlying mechanism. Supplementary exploration was conducted in our study to further elucidate the mechanism behind such association. Consistently, we also observed greatly elevated proinflammatory cytokine level (IL-6, IL-1β, and TNF-α) in addition to increased TREM1 expression in LPS-induced HEnEpCs.

For preliminary investigation, PROMO database was consulted to identify potential transcription factors for TREM1, one of which turned out to be USF2. It caught our interest in that there are studies implying that USF2 overexpression may regulate downstream cytokines in endometritis. For example, Utsunomiya et al. found that USF2 overexpression is closely correlated with the abnormal expression of steroidogenic factor-1 in eutopic endometrial and endometriotic stromal cells [27]. Wu et al. found that it is through USF2 that transcription factor 21 exerts a regulatory effect on downstream steroidogenic factor-1 and estrogen receptor β [12]. We thus hypothesized that USF2 might transcriptionally regulate TREM1 expression in endometritis. Apart from an elevation in USF2 expression, we also observed downregulated TREM1 expression after USF2 knockdown. More importantly, the transcriptional activation of TREM1 by USF2 was confirmed by dual-luciferase reporter and ChIP assays. USF2 has been lately reported to be implicated in the inflammatory response in rheumatoid arthritis, as Hu et al. found that USF2 knockdown markedly decreased the level of proinflammatory cytokines in Th17 cells [28]. Likewise, in our study, USF2 knockdown also led to a significantly reduced concentration of proinflammatory cytokines IL-6, IL-1β, and TNF-α in HEnEpCs under LPS condition. However, overexpressing TREM1 reversed the effect of USF2 knockdown on the inflammatory response of HEnEpCs.

Toll-like receptors, having the ability to recognize pathogens that have invaded, are participants in the activation of innate immune response and the shaping of adaptive immunity [29,30]. Previous study has proposed the possibility of interaction between TREM1 expression and TLR signals in inflammatory mediation [31]. According to the STRING database, TREM1 may bind to TLR2 and TLR4, the upstream of NF-κB signaling pathway [32]. Such binding relationship was evidenced in our study by the results of Co-IP assay. It has also been reported that TREM1 knockdown results in a corresponding reduction in the expression of TLR2/4-NF-κB-pathway-related proteins [13]. Thus, we hypothesized that TREM1 might regulate TLR2/4-NF-κB signaling pathway in LPS-challenged HEnEpCs. Moreover, blockade of TLR2/4-mediated NF-κB signaling pathway has proven to improve pulmonary inflammation in rats with chronic obstructive pulmonary disease [33], play a protective role in ulcerative colitis induced by dextran sulfate sodium [34], suppress acne vulgaris [35], and alleviate LPS-induced inflammation of human periodontal ligament cells [36]. Our results showed that while LPS induced an increase in the expression of TLR2, TLR4, p-NF-κB p65, and p-IκBα, TREM1 interference effectively downregulated their expression in LPS-induced HEnEpCs. Furthermore, notably dampened fluorescence of p-NF-κB p65 was observed in LPS-challenged HEnEpCs after TREM1 knockdown. Additionally, the intervention of TLR2/4-NF-κB signaling pathway rescued TREM1-overexpression-induced inflammation in LPS-stimulated HEnEpCs. These findings verified our hypotheses that USF2 transcriptionally activates TREM1 and that TREM1 regulates TLR2/4-NF-κB signaling pathway in LPS-challenged HEnEpCs.

## Conclusion

It can be concluded from our study that TREM1 transcriptionally activated by USF2 further activates TLR2/4-mediated NF-κB pathway, thereby promoting the inflammatory response in endometritis. Based on previous studies, this study further elucidated the mechanism of TREM1 in LPS-stimulated HEnEpCs and demonstrated the therapeutic potential of targeting TREM1 in the treatment of endometritis.

## Supplementary Material

Supplemental Material

## Data Availability

The raw data supporting the conclusions of this article are available from the corresponding author on reasonable request.
