# Supplementary figures and images for: Upstream stimulatory factor 2 (USF2) induced upregulation of triggering receptor expressed on myeloid cells 1 (TREM1) promotes endometritis by regulating toll-like receptor (TLR) 2/4-nuclear factor-kappaB (NF-κB) signaling pathway
Source: Bioengineered. 2022 Jan 31;13(2):3609–19. doi: 10.1080/21655979.2022.2030619 (PMC8973694; doi:10.1080/21655979.2022.2030619)

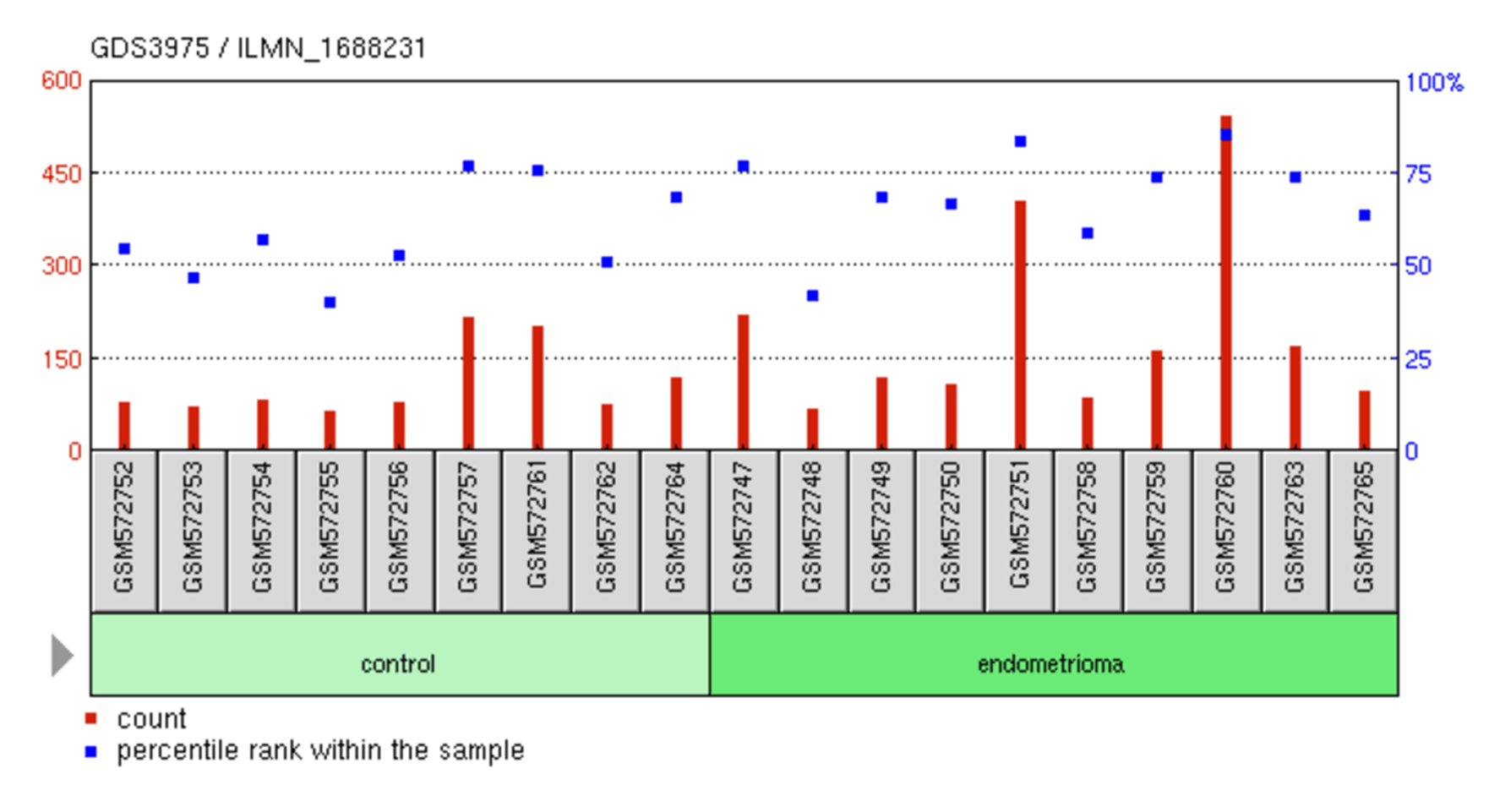

Supplement: Supplemental Material [file KBIE_A_2030619_SM1868.tif]
